# Protective effect of clusterin on oxidative stress-induced cell death of human corneal endothelial cells

**Published:** 2009-12-16

**Authors:** Young Joo Shin, Jeong Hun Kim, Jong Mo Seo, Sang Mok Lee, Joon Young Hyon, Young Suk Yu, Won Ryang Wee

**Affiliations:** 1 Myung Dong St. Mary’s Eye Center, Seoul National University College of Engineering, Seoul, Korea; 2 Department of Ophthalmology, Seoul National University College of Medicine, Seoul National University College of Engineering, Seoul, Korea; 3 Department of Electrical Engineering, Seoul National University College of Engineering, Seoul, Korea

## Abstract

**Purpose:**

To investigate the protective effect of clusterin on oxidative stress-induced cell death of human corneal endothelial cells.

**Methods:**

Human corneal endothelial cells (HCECs) were cultured according to previously published methods. With treatment of various concentrations (0-50 mM) of tert-butyl hydroperoxide (tBHP) or clusterin, reactive oxygen species (ROS) producrion was measured using an oxidationsensitive fluorescent probe and 2′7′-dichlorofluorescin diacetate (DCFH-DA). Cell viability was assayed with a Cell Counting Kit-8.

**Results:**

In HCECs, DCF-DA staining revealed that cells treated with a higher concentration of tBHP had higher fluorescent intensity than cells treated with clusterin, compared to control cells. Clusterin significantly inhibited tBHP-induced ROS production. Cell viability decreased with higher tBHP concentration. Cells treated with clusterin had higher viability than control cells at 5 mM tBHP. Clusterin effectively protected HCECs from ROS-induced cell death.

**Conclusions:**

Our data suggest that clusterin may protect HCECs from oxidative injury-mediated cell death via inhibition of ROS production.

## Introduction

Oxidative stress has been implicated in a wide spectrum of patho-physiological events, including Fuchs’ endothelial dystrophy [1], bullous keratopathy [1,2], and senescence [1]. Although bullous keraopathy patients and Fuchs dystrophy patients often have similar clinical symptoms, their pathophysiologic pathways are not different. The peroxynitrite pathway is induced by oxidative stress in Fuchs’ dystrophy and, on the other side, malonidialdehyde is present as a toxic byproduct of lipid peroxidation induced by reactive oxygen species (ROS) in bullous keratopathy [1]. Tert-butyl hydroperoxide (tBHP) is a ROS generation agent that causes lipid peroxidation [3]. tBHP has been known to decrease cellular proliferative life span and increase the proportion of cells’ positive senescence-associated enzyme activity. Thus, tBHP has been used for the induction of stress-induced premature senescence [3].

Clusterin, a heterodimeric glycoprotein, was first isolated from ram rete testis fluid [4]. Clusterin has been described to attribute to many cellular physiologic functions, including cell–cell interactions [4,5], complement inhibition, lipid transportation, cell survival, and apoptosis [6]. Clusterin, also called apoprotein J, is induced under cytotoxic conditions to protect cells from cytotoxic stress [3,7-12]. it has been reported that clusterin is associated with many degenerative diseases, such as Alzheimer’s disease [13-15] and Huntington's disease [16]. Clusterin has been reported to express in normal corneal endothelilum [17], to increase in corneal endothelium with Fuchs’ endothelial dystrophy [18,19], and to decrease in corneas with bullous keratopthay [18]. It has been predicted that clusterin may play a role in the pathophysiology of these diseased corneas. However, the function of clusterin in human corneal endothelial cells under oxidative stress is not clearly understood.

In the present study, we investigated the protective effect of clusterin on the oxidative stress-induced cell death of human corneal endothelial cells..

## Methods

### Culture of human corneal endothelial cells

Human corneal endothelial cells were cultured according to previously published methods [20,21]. Corneal endothelial cells from the remnant donor tissue after corneal transplantation were harvested attached to Descemet’s membrane on or before the 7th day after death. The ages of the donor tissues were 40 years old (donor A) and 33 years old (donor B). The endothelial cells and Descemet’s membrane complex were incubated for 1 h in 0.02% ethylenediaminetetraacetic acid (EDTA) solution, stirred vigorously with a flame-polished pipette to disrupt cell junctions, centrifuged for 5 min at 3000× g, and seeded onto culture plates coated with FNC coating mix (Athena Enzyme Systems, Baltimore, MD) containing bovine fibronectin (10 μg/ml) and bovine type I collagen (35 μg/ml). The cells were cultured in OptiMem-I media (GIBCO/BRL Life Technologies, Grand Island, NY) supplemented with 8% fetal bovine serum (Cambrex Bio Science, Walkersville, MD), 200 mg/l calcium chloride (Sigma Chemical Co. St. Louis, MO), 0.08% chondroitin sulfate (Sigma Chemical Co.), 20 μg/ml ascorbic acid (Sigma Chemical Co.), 100 μg/ml pituitary extract (Invitrogen, Grand Island, NY), 5 ng/ml epidermal growth factor (Sigma Chemical Co.), 20 ng/ml nerve growth factor (Sigma Chemical Co.), 10 μg/ml gentamicin (Invitrogen, Grand Island, NY), 100 IU/ml penicillin (Cambrex Bio Science), 100 IU/ml streptomycin (Cambrex Bio Science), and 2.5 μg/ml amphotericin (Cambrex Bio Science) under 5% CO_2_. The medium was changed every 2 days. At confluence, the cells were split 1 to 3, and passage 4 cells were used for experiments.

### Immunofluorescence staining

Human corneal endothelial cells (HCECs) cultured on the coverglasses in 12-well plates were washed with phosphate buffered saline (PBS) and fixed for 20 min with 4% paraformaldehyde solution. Cells were permeabilized with 0.1% Triton X-100 for 10 min and blocked with 1% bovine serum albumin for 1 h at room temperature. After washing, cells were incubated with goat polyclonal antibody to the α2 chain of collagen VIII (Santa Cruz Biotechnology, Inc., Santa Cruz, CA) overnight at 4 °C and then washed with PBS. Cells were incubated with FITC-conjugated donkey anti-rabbit IgG antibody (1:100) for 1 h at 37 °C in the dark and were counterstained with Hoechst nuclear staining dye (1:2,000; Molecular Probes, Eugene, OR) according to the manufacturer’s recommendations. After extensive washing with PBS, the slide was mounted in a drop of mounting medium to reduce photobleaching. Negative control staining was performed in parallel with the omission of primary antibodies.

### Purification of clusterin

Clusterin was purified from fresh normal human plasma as previously described [10,11]. Human plasma was precipitated using 12–23% polyethylene glycol (molecular weight 3,350; Sigma Chemical Co.) overnight at 4 °C. This precipitate was dissolved and subjected to diethylamino ethanol-sepharose and heparin-sepharose column chromatography (GE Healthcare Life Sciences, Piscataway, NJ). Clusterin-positive fractions were then applied to a clusterin monoclonal antibody affinity chromatography column. The anti-clusterin monoclonal antibody (1G8) was generated using recombinant human full-length clusterin expressed in *Escherichia coli* as an antigen, and covalently conjugated to cyanogen bromide-activated sepharose 4B (Sigma Chemical Co.). The eluted protein was dialysed against PBS and stored at 280 °C.

### Cytotoxicity test

Cytotoxicity was measured using the Cell Counting Kit-8 (CCK-8) assay (Dojindo Laboratories, Kumamoto, Japan), which is based on the conversion of water-soluble tetrazolium salt, WST-8 [2-(2-methoxy-4-nitrophenyl)-3-(4-nitrophenyl)-5-(2,4-disulfophenyl)-2H-tetrazolium, monosodium salt] to a water-soluble formazan dye upon reduction in the presence of an electron carrier by dehydrogenases [22]. Cells (5×10^4^ cells/ml) were treated with various concentrations (0–50 mM) of tBHP with or without clusterin for 2 h. The cultures in 96-well plates were placed in 100 μl of medium that contained CCK-8 and incubated for 3 h at 37 °C. The absorbance at 450 nm was determined by a multi-plate reader (Lamboda Bio-20; Beckman, Inc., Fullerton, CA). Cell viability was expressed as a percentage of the control (untreated) cells.

### Measurement of intracellular reactive oxygen species formation

Production of ROS was measured using an oxidationsensitive fluorescent probe, 2′7′-dichlorofluorescin diacetate (DCFH-DA, D6665; Sigma-Aldrich, St. Louis, MO) methods, based on the ROS-dependent oxidation of DCFH-DA to DCF. HCECs plated on cover glasses in 12-well plates (1×10^6^/well) were grown in Chen’s media for 72 h. The cells were treated with 0, 0.5, 5, 10, 25, and 50 mM tBHP with or without clusterin (2 μg/ml) at 37 °C for 120 min. Medium was removed and cells were washed by PBS. Then, 200 μl DCFH-DA (10μM) was added for 30 min at 37 °C in the dark. The cells were washed with PBS. Intracellular ROS production was measured using DCFH-DA by the method previously described for the DCF-DA microplate assay [23]. We used a spectrofluorometer (SFM 25; Kontron Instruments) to measure ROS generation by the fluorescence intensity of 10,000 cells in each well at an excitation wavelength of 495 nm and an emission wavelength of 530 nm. Fourteen wells were used for each concentration of tBHP. The fluorescent images were taken by an Olympus microscope with a WU (a near-ultraviolet fluorescence cube) excitation filter (Olympus Corp., Tokyo, Japan).

### Propidium iodide staining

Cell death was assessed by the uptake of the fluorescent exclusion dye PI. PI is impermeable to cells with intact plasma membranes, but when a cell’s integrity becomes compromised, it enters the cells and stains the nucleus. HCECs (1×10^5^ cells/ml) were plated in 12-well plates and were treated with 0–50 mM of tBHP with or without clusterin for 2 h at 37 °C. Cells were washed with PBS and then stained with 10 μg/ml PI (Sigma Chemical Co.) for 30 min at 37 °C. After washing the cells with PBS, the images were obtained using an Olympus microscope with a WU excitation filter (Tokyo, Japan).

### Measurement of caspase-3 activity

The activation of caspase-3 was determined using the Caspase-3/CPP32 Colorimetric Assay Kit (BioVision Inc., Mountain View, CA). The assay is based on the spectrophotometric detection of the chromophore pnitroanilide (pNA) after cleavage from the labeled substrate DEVD (Asp-Glu-Val-Asp)-pNA. Comparison of the absorbance of pNA from an apoptotic sample with an uninduced control allows determination of the fold increase in CPP32 activity. HCECs (5×10^4^ cells) were harvested and caspase-3 activity was determined according to the manufacturer instructions. Absorbance of the chromophore p-nitroanilide produced was measured using a microplate reader (Lamboda Bio-20) at 405 nm. Activity of caspase-3 was expressed relative to the amount of total protein in the cell extracts determined using a BCA protein assay kit. The results were expressed as relative caspase activity.

### Statistical analysis

Data were expressed as the mean±SD from three independent experiments and evaluated using the Kruskal test, followed by the Mann-Whitney test. Significant differences were established at p<0.05.

## Results

### Human corneal endothelial cell culture

**Figure 1:**
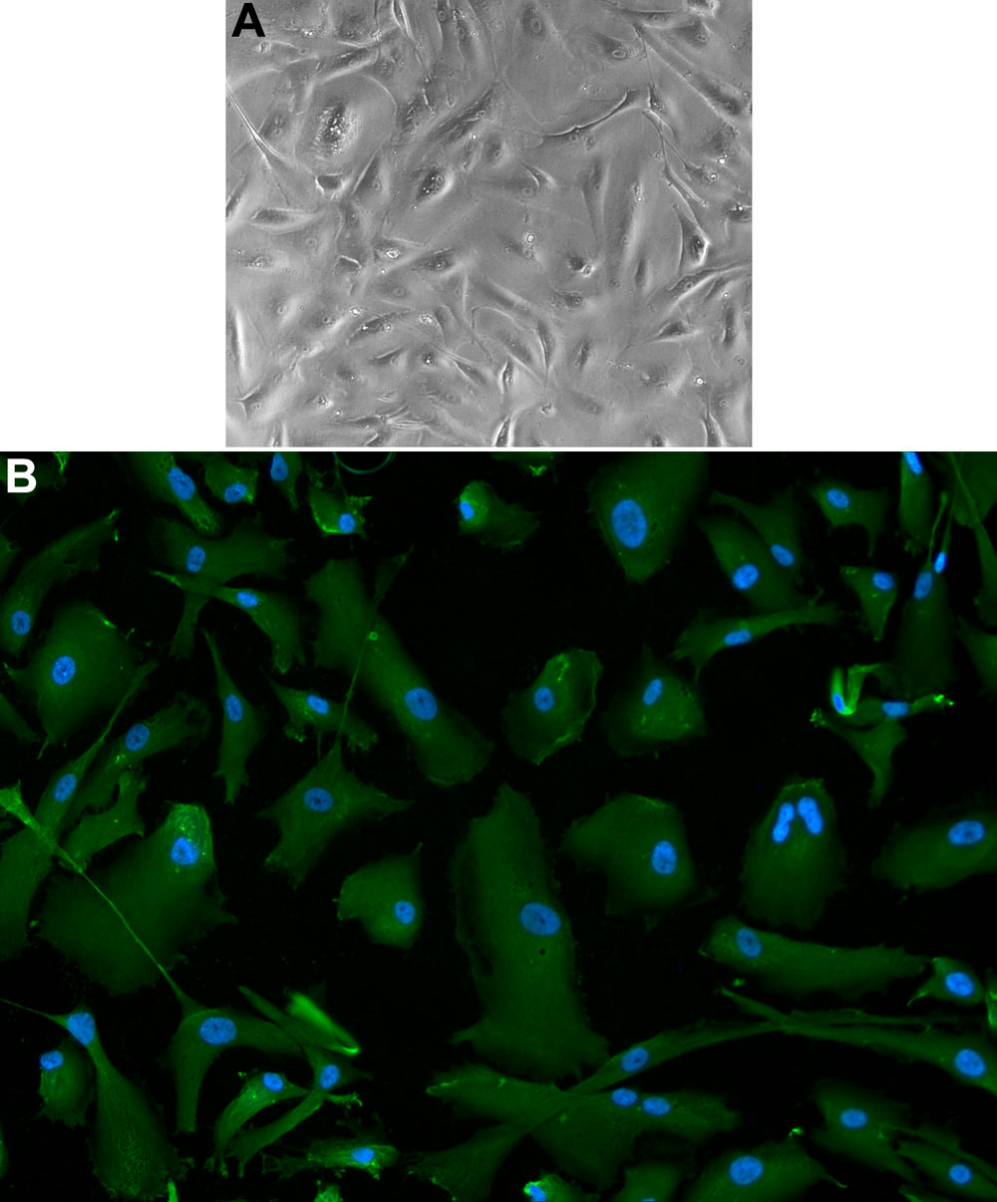
Cultured human corneal endothelial cells.  A: A mosaic pattern is shown. B: Human corneal endothelial cells are positively stained with anti-collagen VIII alpha 2 antibody (red); DAPI nuclear staining is blue.

HCECs were cultured as previously described (Figure 1A) [24]. Corneal endothelial phenotype was verified by the intense positive staining with type VIII collagen alpha 2 monoclonal antibodies (Figure 1B). The green signal within cytoplasm indicated collagen VIII alpha 2 synthesis.

### Cytotoxicity test

**Figure 2:**
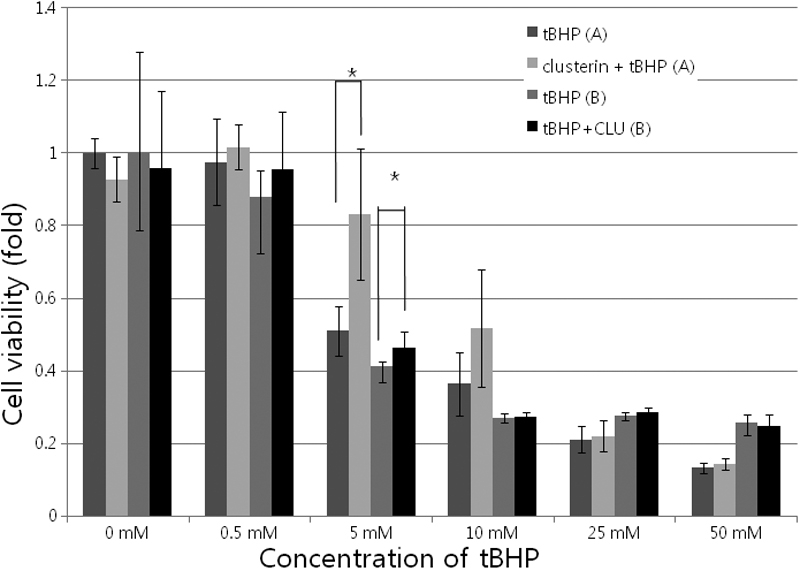
Cytotoxicity test using cell counting kit-8 assay. Various concentrations of tert-Butyl hydroperoxide (tBHP; 0-50 mM) with or without clusterin (2 μg/ml) were used for 2 h in cultured human corneal endothelial cells. tBHP showed a dose-dependent cytotoxic effect on HCECs. At a concentration of 5 mM tBHP, tBHP caused 48.9±6.8% decrease in cell viability and clusterin increased cell viability, compared with the control (p=0.002, Mann-Whitney test). **A** indicates HCECs from a 40-year-old donor. **B** indicates HCECs from a 33-year-old donor.

HCECs were treated with various concentrations of tBHP (0–50 mM) with or without clusterin (2 μg/ml) for 2 h. The effect of tBHP with or without clusterin on cell viability was evaluated by CCK-8 assay. tBHP showed a dose-dependent cytotoxic effect on HCECs (Figure 2). At a concentration of 5 mM tBHP, tBHP caused 48. 9±6.8% decrease in cell viability, and clusterin increased cell viability, compared with the control (p=0.002 [donor A] and 0.032 [donor B], according to the Mann-Whitney test).

### Measurement of intracellular reactive oxygen species formation

**Figure 3:**
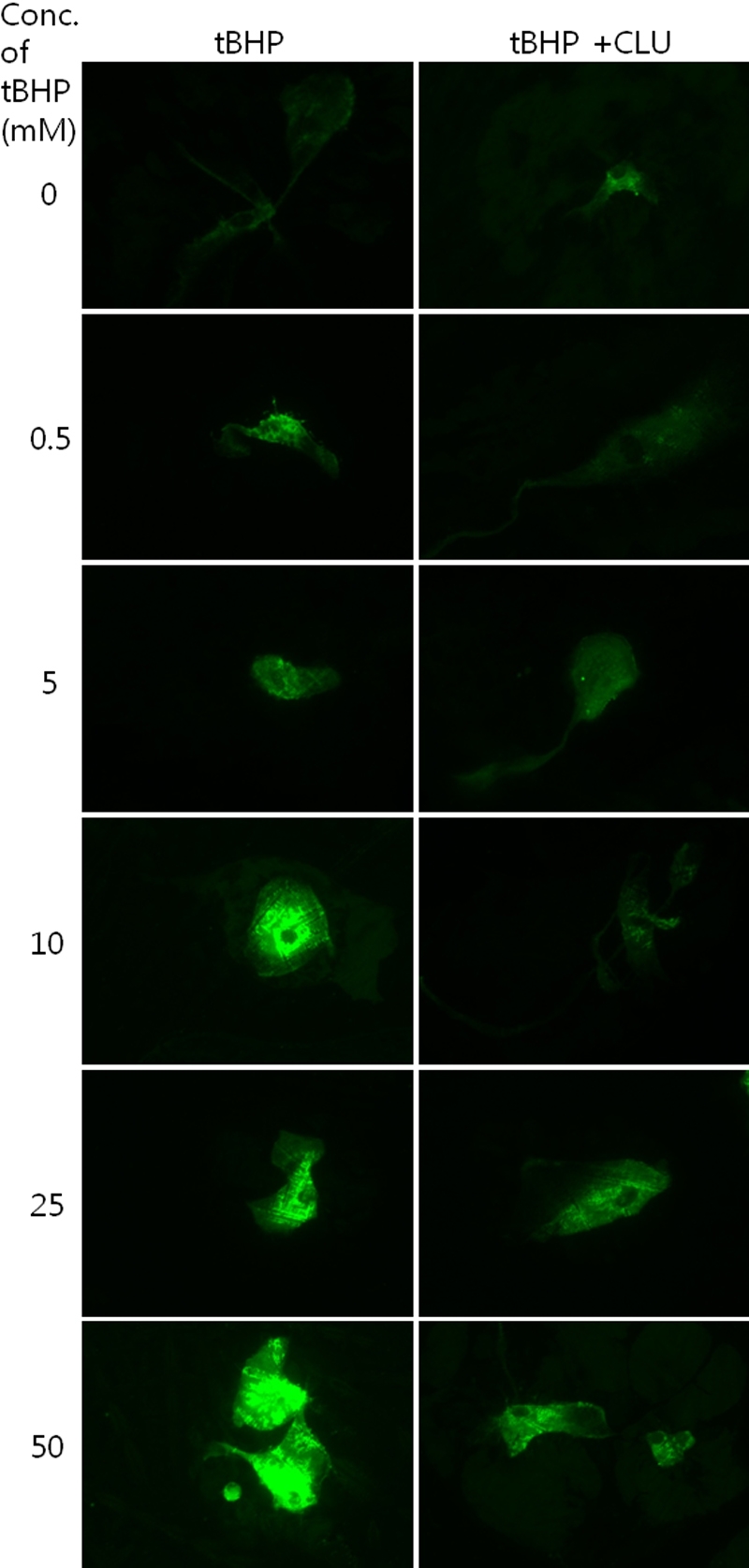
Two-dimensional dichlorofluorescein fluorescent photomicrographs of as human corneal endothelial cells. The cells revealed higher intensity of fluorescence at higher concentrations of tert-Butyl hydroperoxide (tBHP). The clusterin-treated group showed lower dichlorofluorescein (DCF) fluorescence, compared to the control.

**Figure 4:**
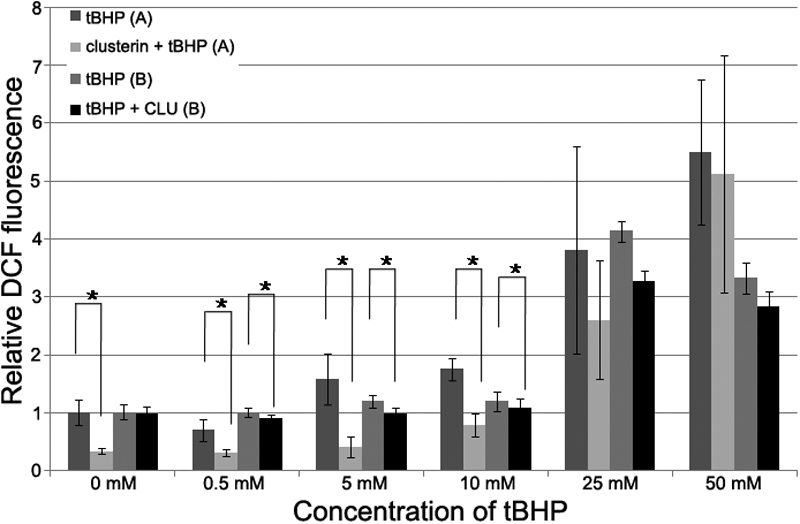
Relative dichlorofluorescein fluorescence. The formation of reactive oxygen species (ROS) was assayed by measuring the fluorescence of dichlorofluorescein (DCF). Each value represents the mean±SD. The asterisk indicates p<0.05, in comparison with the control group. **A** indicates HCECs from a 40-year-old donor. **B** indicates HCECs froma 33-year-old donor.

To estimate the effect clusterin on the changes in intracellular ROS level in cultured HCECs, HCECs were treated with tBHP (0–50 mM) with or without clusterin for 2 h at 37 °C, and the levels of endogenous ROS were measured by DCF fluorescence. As shown in Figure 3, compared with the control group, clusterin-treated groups led to a significant decrease in DCF fluorescence. The results showed that clusterin could decrease the level of intracellular ROS significantly in HCECs (Figure 4; p=0.002, 0.002, 0.004, and 0.002 [donor A], and p=0.841, 0.017, 0.017, and 0.029 [donor B], according to the Mann-Whitney test).

### Propidium iodide staining

**Figure 5:**
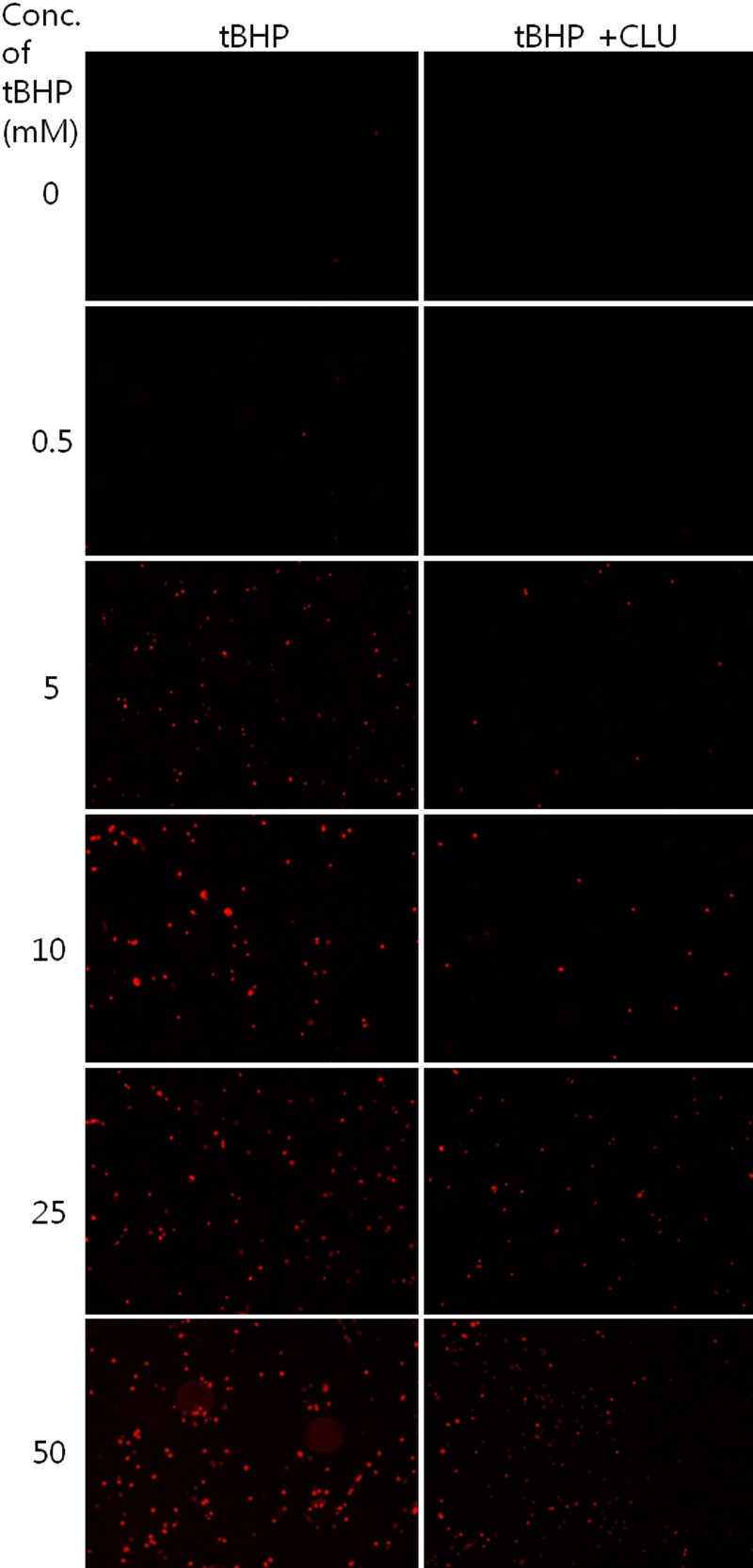
Detection of cell death using propidium iodide staining. The number of propidium iodide (PI)-stained cells increased with higher concentrations of tBHP and decreased in clusterin-treated groups, compared with the control.

To determine whether clusterin inhibits cell death in HCECs, we stained the nuclei with PI. The number of PI-stained cells increased with higher concentrations of tBHP, and decreased in clusterin-treated groups, compared with the control (Figure 5).

### Effects of clusterin in changing caspase-3 activity in human corneal endothelial cells

**Figure 6:**
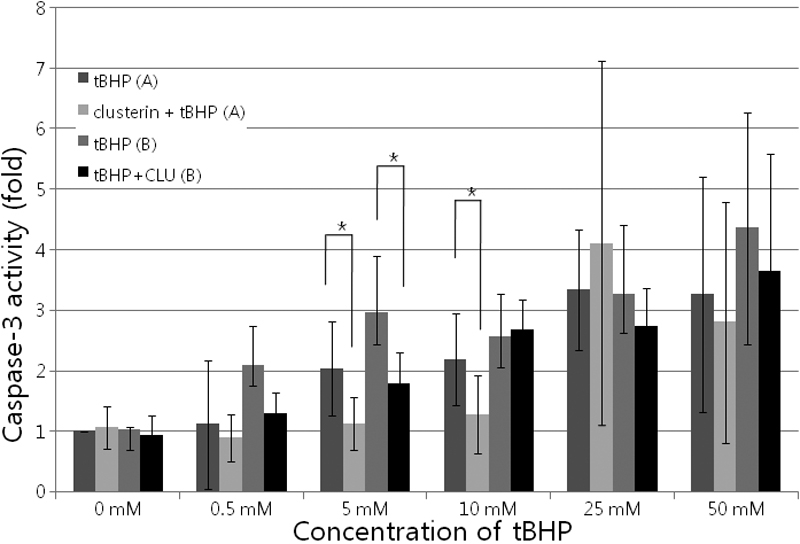
Effects of clusterin on the caspase-3 activity in cultured human corneal endothelial cells. Activation of caspase-3 was determined by spectrophotometric detection of the chromophore p-nitroanilide (pNA). Each value represents the mean±SD. The asterisk indicates a p<0.05, in comparison with the control group. **A** indicates human corneal endothelial cells (HCECs) from a 40-year-old donor. **B** indicates HCECs from a 33-year-old donor.

Since it is well known that the caspase cascade is activated during apoptosis, we examined the effects of clusterin on caspase-3 activity. As shown in Figure 6, HCECs treated with tBHP with or without clusterin for 2 h, showed a significant decrease in caspase-3 activity, compared with the non-treated control group. At a concentration of 5 mM tBHP, tBHP caused an increase in caspase-3 activity, and clusterin decreased caspase-3 activity, compared with the control (p=0.001 [donor A] and 0.0009 [donor B], according to the Mann-Whitney test). These results indicated that clusterin attenuated the apoptosis of HCECs via suppression of the caspase cascade.

## Discussion

Clusterin is expressed in a broad range of eye tissues [25,26], including the cornea and conjunctiva in human. It has been demonstrated that uniform, punctate clusterin staining was present in a normal corneal endothelium [18]. Clusterin has been described as being overexpressed in corneal diseases, including severe ocular surface diseases [27-29] and Fuchs’ endothelial dystrophy [30-32]. However, there has been no study that investigates the effect of clusterin on HCECs. In the present study, we treated HCECs with various concentrations of tBHP, with or without clusterin. tBHP was used as an ROS-generating agent that could induce oxidative stress. tBHP is known to be able to induce stress-induced premature senescence [3]. In addition, tBHP damages DNA, resulting in cell death [33,34]. Fuchs’ endothelial dystrophy and bullous keratopathy have been reported to be related to oxidative stress [1,2,35] and apoptosis [36,37].

In the present study, clusterin decreased intracellular ROS induced by tBHP in cultured HCECs. DCF-DA staining revealed that cells treated with higher concentrations of tBHP had higher fluorescence, and that cells treated with clusterin had lower fluorescent intensity, compared to control cells. Clusterin significantly inhibited tBHP-induced ROS production. These results are compatible with previously described data [3]. Cell viability decreased with higher tBHP concentration, and cells treated with clusterin had higher viability, compared to the control at 5mM tBHP. Clusterin effectively protected HCECs from ROS-induced cell death. PI staining showed that clusterin decreased tBHP-induced cell death (Figure 5). Clusterin appears to be a part of the cellular response to oxidative stress [38]. It has been suggested that secretory clusterin may act as an extracellular molecular chaperone, scavenging extracellular misfolded or denatured proteins that can be produced following stress-induced injury [39]. Additionally, it has been proposed that clusterin has antioxidant properties and is capable of protecting cells from apoptosis induced by ROS [40]. The redox difference is shown in the 0 mM concentration of tBHP in Figure 3. Clusterin decreased redox state, even in normal control cells. This should be investigated in further, molecular-based studies.

The viability results obtained by propidium iodide staining did not correlate well with those obtained by cytotoxicity testing. In general, both the PI cytotoxicity assay and CCK-8 assay have been widely used for determining cell viability. However, the mechanisms are different. CCK-8 assay is based on the ability of viable mitochondria to convert water-soluble tetrazolium salt, WST-8, to a water-soluble formazan dye, upon reduction in the presence of an electron carrier by dehydrogenases [22]. As for PI, it does not penetrate the intact plasma membranes that are either viable or in the early stages of apoptosis. It enters the cells and stainstheir nuclei in the later stages of apoptosis, or when the cells are already dead.

Caspases are the cysteine proteases that are crucial for the process of apoptosis. Caspase 3 functions in the execution of apoptosis cascades. Increased caspase 3 gene expression or activity is considered to be one of the hallmarks of apoptosis induction [41,42]. In the present study HCECs treated with clusterin showed a significant decrease in caspase-3 activity, compared with the non-treated control group. These results indicated that clusterin did attenuate the apoptosis of HCECs via suppression of the caspase cascade.

This study is the first study to investigate the effect of clusterin on HCECs under oxidative stress. The clusterin-treated group showed high cell viability, low ROS generation, and low cell apoptosis in HCECs, compared with the control. Although transgenic Sa OS and U2 OS cell lines adapted to high intracellular clusterin levels are sensitive to genotoxic and oxidative stress [43], clusterin has been reported to have a protective effect against oxidative stress in various cell lines [7,8,10,44,45], as well as against ischemic tight junction protein loss and human retinal endothelial cell death [11]. In the present study, we used exogenous clusterin purified from serum. Clusterin in serum is the glycosylated secretary form (sCLU) [46,47]. It has been demonstrated that the increased level of the secreted form and the disappearance of the nuclear unglycosylated one (nCLU) are directly connected to increased cell survival [12]. Previous studies have indicated a dose-dependent effect of clusterin on the survival of the cultured cells [48,49].

In conclusion, our data suggest that clusterin could protect HCECsfrom oxidative injury-mediated cell death via inhibition of ROS production. Exogenous clusterin may be useful as a protective agent during intraocular surgery in old patients and patients with Fuchs endothelial dystrophy or bullous keratpathy. In addition, it may be useful as a pretreatment agent against corneal endothelial injury occurred during DMEK, DSAEK, and cultured cell transplantation.
